# College of Physicians and Surgeons of the University of the State of New York

**Published:** 1848-08

**Authors:** 


*College of Physicians and Surgeons of the University of the State of New York.*

Since the publication of the advertisement of this Institution in the last number of this Journal, some changes have been made in the arrangements for the next course of lectures. The session will commence on the 30th of October, instead of the 13th, and the preliminary course will occupy the whole of October, one month before the regular lectures commence [see advertisement in this number.] Charles E. Isaacs, M. D., has also been appointed demonstrator of anatomy; a gentleman eminently fitted for the duties of that office.

